# Theory of Mind in Borderline Personality Disorder: A Possible Endophenotypic Factor?

**DOI:** 10.3390/ijerph18063193

**Published:** 2021-03-19

**Authors:** Esther Ortega-Díaz, Jonatan García-Campos, Alejandro Moya-Martínez, Clara Ramírez-Cremades, José M. Rico-Gomis, Carlos Cuesta-Moreno, Antonio Palazón-Bru, Gabriel Estan-Cerezo, José A. Piqueras, Jesús Rodríguez-Marín

**Affiliations:** 1 Department of Psychiatry, General University Hospital of Elche, 03203 Elche, Spain; estherapeuta@icloud.com (E.O.-D.); c.ramirezcrem@gmail.com (C.R.-C.); chemarir@hotmail.com (J.M.R.-G.); carlos.cuesta93@gmail.com (C.C.-M.); 2 Department of Behavioral and Health Sciences, Miguel Hernández University of Elche, San Juan de Alicante, 03202 Elche, Spain; 3 Biostatistics Unit, General University Hospital of Elche, Elche-FISABIO, 03203 Elche, Spain; bioestadistica_elx@gva.es; 4 Center for Operational Research, Miguel Hernández University of Elche, 03202 Elche, Spain; 5 Department of Clinical Medicine, Miguel Hernández University of Elche, San Juan de Alicante, 03202 Elche, Spain; apalazon@umh.es; 6 Department of Investigation, General University Hospital of Elche, Elche-FISABIO, 03203 Elche, Spain; gabrielestan@gmail.com; 7 Department of Health Psychology, Faculty of Social and Health Sciences, Campus of San Juan de Alicante, Miguel Hernandez University (UMH), 03202 Elche, Spain; rod.marin@umh.es; 8 Center for Applied Psychology, Campus of Elche, Miguel Hernandez University (UMH), 03202 Elche, Spain

**Keywords:** borderline personality disorder, theory of mind, mentalization, family, endophenotypic marker

## Abstract

The purpose of this study is to examine whether theory of mind (ToM) is an endophenotypic marker of borderline personality disorder (BPD), thus constituting an etiopathogenic factor of the disease. This would suggest familial vulnerability to BPD. This was a case-control study involving 146 individuals with 57 BPD patients, 32 first-degree relatives, and 57 controls (median age of BPD and control = 33.4 years; relatives = 52.9 years; BPD females and controls = 91.2%; female relatives = 62.5%). All the participants completed the Spanish version of the Movie for the Assessment of Social Cognition test to evaluate the ToM subclassification: interpretation of emotions, thoughts and intentions. BPD patients and their healthy first-degree relatives exhibited significant deficits in the correct interpretation of emotions and intentions compared to healthy controls. Both patients with BPD and their healthy first-degree relatives exhibited significant deficits in ToM, which suggests that it may be an etiopathogenic factor of BPD, and ToM (interpretation of emotions, thoughts and intentions) is a possible endophenotypic marker of BPD, suggesting a genetic predisposition to the disorder. Therefore, ToM could be considered as an indicator for the early detection of the disorder of and intervention for BPD.

## 1. Introduction

Borderline personality disorder (BPD) is a serious psychiatric illness that affects 5.9% of the population [1]. It is considered to be the most prevalent personality disorder in the clinical setting, accounting for 10% of psychiatric outpatients and 15–20% of hospitalized patients [2]. Despite its high prevalence, however, BPD is often underdiagnosed [3].

Patients with BPD exhibit hypersensitivity in social situations [4,5], experiencing an inordinate fear of abandonment or disproportionate anger in separations or changes of plans [4]. Several studies have associated BPD with impaired social cognition [6,7,8,9,10,11,12]. Regarding theory of mind (ToM), patients with BPD have a normal ability to recognize facial emotions [13,14,15] and isolated prosodic features [16,17] and even to make attributions of the intentions of others [18]; however, they find deficits in recognizing facial emotions and integrated prosodic features [6,9,17,19]. Then, affective ToM is preserved, while cognitive ToM presents a deficit [19]. Patients with BPD make more perverse interpretations of others compared to controls [20]. In the study by Wagner and Linehan [21], they report that they interpret neutral facial expressions in a more negative way. In the study by Domes et al. [22], they showed a bias towards the perception of anger. In the studies of Sharp et al. [9] and Ortega et al. [23], overmentalization errors were seen in adolescent patients with borderline traits and in adult patients with BPD, respectively. According to Green et al. [11], “social cognition refers to the mental operations underlying social interactions, which include processes involved in perceiving, interpreting, and generating responses to the intentions, dispositions, and behaviors of others”. One of the most important components of social cognition is theory of mind (ToM), a socio-cognitive construction defined as the ability to attribute mental states to oneself and to others [12]. ToM skills are necessary to forge and maintain interpersonal relationships [24,25,26]. However, few studies have described relationship disturbances in BPD [27,28,29]. The relational style of BPD patients has been reported to be the most suitable marker for diagnosis [30].

If a deterioration in ToM is considered a state type, it is regarded as a secondary symptom. However, if it is considered a trait, it constitutes primary deterioration, an etiopathogenic factor of the disease—in other words, an early marker of the disease. This can establish a new paradigm for testing hypotheses on the nature, onset and evolution of BPD. Studies on relatives can help to address if an alteration is found only in subjects with the diagnosis, or if it is also present in relatives. The objective is to estimate the effect of family influences on the disorder and the possible corresponding endophenotype or intermediate phenotype [31]. One of the methods to verify this is by studying a population with a significant genetic load, such as first-degree relatives. Studying the possible alterations in social cognition through ToM might provide valuable insights into their effects on BPD patients and first-degree relatives [31]. Deficits in ToM have been investigated in studies with relatives in various psychiatric diagnoses, including studies with relatives in bipolar disorder [32,33,34] or schizophrenia [35,36,37,38,39,40,41,42]. Although there was a meta-analysis on ToM and BPD in 2017 [19], almost all studies on relatives of BPD have been based on interviews and self-report measures of diagnoses and symptoms, only two studies used neuropsychological measures [43,44] and, to the best of our knowledge, only two studies have investigated social cognition in first-degree relatives of BPD patients [23,45]; none have examined ToM or relational style through an audiovisual test. Gulamani [45] reports that probands and relatives showed a stronger tendency than controls to misinterpret sad and fearful faces, concluding that biases associated with the perception of sad and fearful faces are found in both patients and family members. However, one of the limitations of his study is the use of static images, since less information is provided than in tests that use dynamic faces [46], and the use of ecologically valid tests to overcome this limitation is recommended. The present study examines ToM using the audiovisual scale Movie for the Assessment of Social Cognition (MASC) [47], evaluating the ability of patients with BPD to identify emotions, thoughts and intentions, compared to their first-degree biological relatives and the healthy population.

The aim of this study was to examine whether theory of mind is an endophenotypic marker of BPD, suggesting genetic vulnerability of patients, and thus being an indicator for the early detection of the disease. We applied the Spanish version of the Movie for the Assessment of Social Cognition (MASC) scale [47] using the ToM subclassification, in which the relational style is analyzed through the interpretation of emotions, thoughts and intentions.

## 2. Materials and Methods

### 2.1. Participants

The study population consisted of BPD patients, healthy first-degree relatives and the general population with no mental pathologies recruited in the Department of Health 20 of the General University Hospital of Elche (Alicante, Valencian Community, Spain).

### 2.2. Study Design and Participants

This is a case-control study involving 146 individuals, 57 of whom were BPD patients, 32 were healthy first-degree relatives, and 57 were controls. Patients registered under BDP ICD-9-CM code 301.83 and ICD-10-CM code F60.3 were selected from the database of the General University Hospital of Elche and recruited between July 2018 and March 2019. Their diagnoses were confirmed according to the DSM-5 diagnostic criteria [4]. The exclusion criteria were an age under 18 years, diagnosis of intellectual disability, associated severe mental disorders, residence in another autonomous community and incarceration. First-degree relatives were parents or children of the patients. Relatives who were under 18 years of age or had any psychiatric disorder were excluded. The reason that relatives under 18 years of age were excluded is because ToM can vary in children, where it is not yet formed. In addition, until the age of 18 the diagnosis of BPD is not made, thus recruiting a possible relative who will develop BPD was avoided to minimize creating a confounding factor. Only one relative of each BPD patient participated. A total of 43 family members volunteered, of whom only 32 were selected—11 were excluded for presenting mental pathologies. In total, 25% of the total were excluded; this is consistent with data on psychiatric comorbidity in BPD relatives [48]. BPD patients and their relatives were contacted by telephone and were given an appointment at the hospital if they were willing to participate in the study. Controls were recruited among individuals accompanying patients to the surgery, internal medicine, trauma, neurology, and obstetric services. They were matched with the BPD patients in terms of age, sex and education level. Possible psychiatric disorders were ruled out for relatives and controls using the International Neuropsychiatric Interview [49].

### 2.3. Variables and Measures

Social cognition was evaluated using the scale “Movie for Assessment of Social Cognition” (MASC), the Spanish version [47]. The MASC scale was created in Germany in 2006 by Dziobek et al. [50]. The MASC is an ecological test based on a video presenting various social situations amongst four characters and is composed of 45 questions, with four possible answers each, in which only one answer out of the four is correct. Results are measured regarding the feelings, thoughts and intentions of the characters, which are asked during the video. The video stops after each scene and a multiple choice question appears with four alternatives that must be answered in a maximum time of 30 s.

The score considers the number of hits and misses, and the subtype of the misses is analyzed [49]. The maximum score is 45 points. Of the 45 items, we found 15 items that measure the correct interpretation of emotions, 4 items that measure the interpretation of thoughts, 14 items that measure the interpretation of intentions, and 12 items that measure several of these at the same time. The lower the number of hits, the more serious the deterioration. The MASC has high interrater reliability (intraclass correlation coefficient = 0.99) and high test–retest reliability [50] (*r* = 0.97). The MASC subscales have a high degree of internal consistency (emotions subscale: Cronbach’s α = 0.62; thoughts subscale: Cronbach’s α = 0.55; intentions subscale: Cronbach’s α = 0.71).

A series of sociodemographic data were also collected: gender, age, education level (primary, secondary or tertiary), civil status (single, married/with a stable partner and widow/widower), employment (active, unemployed, on sick leave, retired and student) and coexistence (own family, family of origin and single).

### 2.4. Sample Size

Statistical power was calculated using the previous work of Ortega-Díaz et al. [23] in which different variables were collected for the study using unbalanced one-way analysis of variance (ANOVA) for the BPD patient (*n* = 57), relative (*n* = 32) and control (*n* = 57) groups. The effect sizes were calculated for the three subscores (emotions, intentions and thoughts). The largest size obtained was that of the intentions variable (0.30; classified on the Cohen scale as medium). Setting the level of significance to 95%, we obtained 99% statistical power.

### 2.5. Statistical Analyses

Qualitative variables were expressed as absolute and relative frequencies, while quantitative variables were expressed as means and standard deviations. The groups’ homogeneity was assessed using Pearson’s chi-squared test and ANOVA. Differences in the subscales between the three groups were evaluated using ANOVA. The Bonferroni test was used for multiple comparisons between the factors. Multivariate regression analysis was performed to investigate associations between factors in the study groups. All analyses were performed at a 5% level of significance, and the confidence interval (CI) for each parameter was calculated. The statistical packages used were IBM SPSS Statistics 25 and R 3.5.1.

### 2.6. Ethical Considerations

This study was conducted in accordance with the latest version of the Declaration of Helsinki. The study was approved by the Ethics Committee and the Research Commission of General University Hospital of Elche, Elche, Alicante, Spain (25 June 2018 and 27 June 2018, respectively). All participants were informed of the study’s aims and methods and signed informed consent forms.

## 3. Results

In this study, information was collected from 154 participants; 91.2% of the participants in the BPD and control groups were females, as were 62.5% of the BPD patients’ relatives. We found a significant difference in at least one of the three groups (*p* < 0.001). All *p*-values reported in Table 1 were interpreted as significant differences in the comparison of at least one of the three groups. The mean age of the participants was 33.4 years in the BPD and control groups and 52.9 years in the relative group (*p* < 0.001). Secondary education was the most frequent education level in the BPD and control groups, while the education level in the relative group was lower (*p* = 0.027). Most participants in the BPD group were single (*p* = 0.006). The BPD group had the most unemployed participants (*p* < 0.001), as well as the most participants living with their families of origin (*p* = 0.004) (Table 1).

Significant differences were found between the control and relative groups with respect to the MASC emotions and thoughts subscores (*p* = 0.009 and *p* = 0.03, respectively). Significant differences were also found in the MASC intentions subscores between the control group and the relative (*p* = 0.013) and BPD (*p* = 0.012) groups (Table 2 and Table 3). The univariate analysis revealed statistically significant differences between the three groups in the MASC emotions (*p* = 0.009) and intentions subscores (*p* = 0.003) (Table 2).

As we can see in Table 1, there is a clear difference in education between controls and BPD vs. relatives. For this reason, a stratification of this variable was carried out in the multivariate analyses. This interaction was only found to be significant when studying the subscore intentions (Table 4). For the evaluation of ToM, we used multivariate linear regression models that included the education level, sex, age, marital status, employment, coexistence and group variables (Table 4 and Table 5).

Table 5 therefore shows the results of the multivariate comparison between the groups for the emotions and thoughts variables. After inputting all the study variables into the model, we obtained significant differences only with the education level, civil status and age.

The education level and group variables were found to influence ToM (Table 5). The relative group had a lower probability of obtaining higher MASC scores than the control group. For example, the probability of the relative group obtaining a higher emotions subscore than the control group was 1.11 times lower. The BPD group was 78% less likely to achieve a higher emotions subscore than the control group. All probabilities were adjusted for education, as it was found that the higher the education level, the higher the probability of obtaining a higher emotions subscore (122% for high school or professional education and 167% higher for university education). 

Conversely, no statistically significant differences in the thoughts subscore were found between the three groups. However, participants with secondary education were 44% more likely to interpret thoughts correctly than participants with primary education (*p* = 0.006). Moreover, single participants had a 67% lower probability of interpreting thoughts than participants who were married or had a stable partner (*p* < 0.001). All study participants had a 2% lower probability of obtaining a higher thoughts score for each increase of one year in age (*p* = 0.013).

The normality, such as heterogeneity and homogeneity, of the residuals was evaluated in all models. Normality was verified for all but the thoughts subscore model, where the residuals were not normally distributed (*p* < 0.001). However, given that the Q-Q plot showed normal distribution, normality was assumed for this subscore as well.

Similar results, albeit with different levels of probability, were obtained for the intensions subscore (Table 4). As it has been proven, there was an interaction of intentions and educational level; therefore, this will be analyzed separately by each educational level. For primary education level, the relative group was 140% less likely and the BPD group was 112% less likely to obtain a higher score than the control group. Moreover, participants living with their own families were 140% less likely to accurately identify intentions than participants living with their family of origin. Amongst the people who have studied secondary school, the BPD patients are 127% less likely to obtain a higher score than the reference group (control group). For secondary educational level people, only BPD patients obtained a probability of 1.27 times less than the controls to obtain a higher score in the subscore intentions. Lastly, with the smaller size of people that were analyzed (*n* = 19), we were less likely to obtain higher scores in intentions in the relatives (220%) and BPD (195%) groups compared to the controls. People with secondary education and people with university education did not influence the coexistence variable.

## 4. Discussion

In this study, we investigated ToM in BPD patients, their healthy first-degree relatives, and healthy controls. Our results show that both BPD patients and their first-degree relatives exhibit significant deficits in ToM, in terms of their relational style, particularly related to the identification of emotions and intentions. This suggests a possible endophenotypic marker—that is, a genetic predisposition to BPD. In fact, our results meet the five criteria established in psychiatry to be considered as an endophenotypic marker [51]. We consider this to be a pioneering, albeit preliminary, advance.

### 4.1. Comparison with the Literature

Few studies on healthy first-degree relatives of BPD patients have been conducted. It has been previously described that BPD patients’ parents exhibit greater response latency when planning a task; however, unlike BPD patients, they do not exhibit impairment of executive functions [43]. Therefore, executive functions do not appear to be markers of familial vulnerability to BPD. Ruocco et al. found that first-degree relatives of BPD patients have greater difficulties in attention and memory than the general population [52]. The authors also reported that BPD patients’ parents exhibit greater response latency when planning a task; however, unlike BPD patients, they do not exhibit impairment of executive functions [52]. Therefore, according to the authors, executive functions do not appear to be markers of familial vulnerability to BPD. Another study by Ruocco et al. [44] found that BPD patients exhibited deficits in response inhibition tests and that unaffected biological sisters obtained very similar scores. These findings suggest that these deficits may be hereditary, regardless of diagnosis, and are thus possible endophenotypic neuropsychological markers of BPD. Another study investigating familial coaggregation of BPD suggested that common family factors, specifically affective disturbance and impulsivity, contribute to BPD [53]. Silverman et al. [54] showed that the risk of affective and impulsive personality disorder traits associated with BPD is higher in patients’ relatives than in controls. In line with these findings, another study reported that the rates of mood and personality disorders are higher in BPD patients’ relatives than in the general population [55]. Moreover, it has been shown that relatives of BPD patients have a significantly higher risk of BPD than relatives of controls [56]. Another study reported high rates of borderline and avoidant personality disorders in relatives of adolescents with BPD, even after adjusting for comorbidity [57].

The most frequent psychiatric diagnoses in BPD patients and relatives are major depression, substance use disorders, post-traumatic stress disorder, anxiety disorders and avoidant personality disorder [58]. Ruocco et al. found evidence of family aggregation in impulsivity, emotional dysregulation, attention difficulties and neuroticism and consciousness traits [58]. Another study reported that among borderline patients, 38.3% had a first-degree relative with depression, 25.5% had a relative with pathological mood swings, and 23.4% had a relative with “eccentric or peculiar behavior” [48].

To the best of our knowledge, two studies have analyzed social cognition in first-degree relatives of patients with BPD [23,45], and none used the MASC subscales to assess ToM through the interpretation of emotions, thoughts and intentions. This classification is particularly relevant, with direct implications for the analysis of patients’ relational styles, the most suitable marker for the diagnosis of BPD [30]. However, it has been studied in other mental pathologies. In one study, relatives of patients with schizophrenia did not show significant deficits in social cognition, but they did show lower performance than the general population [33]. These results are in line with those of Mostag et al. [35], who measured ToM in relatives of patients with schizophrenia using the MASC test and empathy using the Interpersonal Reactivity Index and found significant differences in ToM. Janssen et al. [36] compared patients with schizophrenia in remission with first-degree relatives and a healthy population without a history of mental illness and found that patients had a greater deficit in ToM tasks than the healthy population and that first-degree relatives obtained intermediate scores. A meta-analysis of first-degree relatives of patients with schizophrenia showed that first-degree relatives perform worse than controls in some areas of social cognition, such as theory of mind, emotional processing and social perception [37]. Other studies have also shown that first-degree relatives of patients with schizophrenia exhibit deficits in social cognition compared to the general population [38,39,40,41,59].

In bipolar disorder, a study showed that family members evaluated with verbal component tests exhibited alterations in theory of mind [33]. In this disorder, social cognition impairment is considered a trait characteristic—that is, constant and not manifesting only during periods of crisis—and is an endophenotypic marker, as it is found in populations with a high genetic load, such as first-degree relatives of patients with bipolar disorder [32].

In this study, we used the MASC subscales to evaluate ToM through the interpretation of emotions, thoughts and intentions. We found significant alterations both in BPD patients and in their first-degree relatives in components associated with their relational styles, such as the interpretation of emotions and intentions, compared to healthy controls.

According to Reynolds et al. [33], ToM verbal deficits in family members with bipolar disorder may be a potential candidate for an odophenotype of bipolar disorder. Similarly, findings in relatives with bipolar disorder support the idea that an alteration in ToM processing may act as an intermediate phenotype of bipolar disorder [34].

### 4.2. Strengths and Limitations

The main strength of our study is this is the first to identify ToM, especially with regard to the deficit in identifying emotions and intentions observed in both patients and their relatives, as a possible endophenotype of BPD, and this is a pioneering advance, although this knowledge should be developed by opening new future lines of research in which other types of techniques such as biochemical markers or neuroimaging techniques are used that could deepen this finding.

To minimize selection bias, the controls were matched with the BPD patients in terms of sex, age and education level. To minimize information bias and increase the reliability of our results, the sample collection was performed by a single professional. We used the MASC scale, a naturalistic scale capable of detecting minute changes, which has been internationally validated [50]. Finally, using multivariate models, we were able to minimize confounding bias. This was confirmed by the fact that significant results in the bivariate analysis lost their statistical significance after adjusting for other factors. One of the limitations of the analysis in the stratification by educational level was found in the subscore intentions. In this subscore, the sample of university educational level in the three study groups and secondary level in the relatives are sample sizes that are too small to draw conclusions. To solve these differences in educational level, the stratification of all substudents by the variable level of education has been used and favorable results have only been obtained in the intention subscore in patients and family members compared to controls.

Another limitation of this study is that only one ToM test was used; however, it was selected following the indications of the meta-analysis on ToM and borderline personality disorder (BDP) carried out in 2018 [19]. In this meta-analysis, it is stated that tasks with a higher level of complexity and high ecological validity such as the MASC scale detect alteration in ToM in patients with BPD [10,17]. Future studies should employ a multimethod and multimeasure approach. Another limitation is that we have not been able to make a discussion including other studies carried out on BPD relatives, so a discussion has been carried out with other mental pathologies.

Another limitation would be the number of family members with secondary and university educations collected in our sample; more evidence would be needed to verify whether education is a confounding factor in the analysis of intentions. Regarding this issue, the results of Rodríguez’s study [42] indicate that scores in ToM are not affected by educational level. This study deals with relatives of patients with schizophrenia. They performed an ANOVA to compare the scores of the patients grouped into three educational levels (primary, secondary and higher), in each of the dimensions of social cognition evaluated. There were no differences in the comparisons according to the level of education (*p* > 0.10 in all ANOVA). This result allows us to conclude that the scores in social cognition are not affected by the educational level of the patients.

### 4.3. Implications for Clinical Practice and Research

The presence of significant deficits in ToM, specifically related to the identification of emotions and intentions, in both BPD patients and their first-degree relatives suggests a probable endophenotypic marker—that is, a continuous trait characteristic that extends beyond periods of crisis—may indicate a genetic predisposition to the disorder. Identifying an etiopathogenic factor of the disease raises new questions not only about its nature, but also about its onset and evolution and provides a marker for the early detection of this disease, as well as for rehabilitation interventions and pharmacological treatment.

The same as Santos et al. [32] and Renolds et al. [33], we believe that the deficits found in the ToM of the relatives in our study could indicate that ToM is an intermediate phenotype of BPD. This study supports the beginning for possible lines of research that address this issue through imaging and genetic tests [34] and verify where a multimethod and multimeasure approach should be employed. The possibility of establishing a new paradigm in psychiatry that seeks specific biochemical markers that help in the early identification of pathologies, leading to a new, more evolved psychopathology and leaving behind the exclusive use of phenomenology, has dominated the field since the 19th century.

This new paradigm envisions the identification of symptoms indiscernible to the clinical eye. In this sense, the study of ToM and its deficits as a prodromal and even premorbid element of the disease offers new possibilities for its diagnosis and treatment at the clinical level.

Regarding interventions, BPD patients who have been trained in the management of adaptive strategies exhibit diminished emotional and physiological responses [60]. The identification of ToM deficits in patients’ relatives means that this element could be incorporated in such training.

Although our results meet the five criteria required to be considered as an endophenotype marker in psychiatry, it has not been considered that this deficit in ToM could be due to family factors; future studies should delve into this aspect.

The contribution of nongenetic or environmental variables cannot be ruled out, if not with a twin design, since family members share not only part of the genetic load but the common learning history, culture, family relationships, etc., and it would even be interesting to know to what extent the environment or genotypes can modify the development of phenotypes, in this case speaking of the influence of epigenetics [61].

## 5. Conclusions

In conclusion, this study shows that both BPD patients and their healthy first-degree relatives exhibit significant deficits in ToM, suggesting a possible endophenotypic marker—that is, a genetic predisposition to the disorder.

## Figures and Tables

**Table 1:** Considered sociodemographic factors amongst borderline personality disorder (BPD) patients, first-degree relatives and controls.

Variable	Controls	Relatives	BPD	*p*-Value
	*n* = 57	*n* = 32	*n* = 57	
	$n\;(\%)/\bar{X}$ ± SD	$n\;(\%)/\bar{X}$ ± SD	$n\;(\%)/\bar{X}$ ± SD	
Age (years)	33.4 ± 10.7	52.9 ± 16.3	33.4 ± 10.7	**<0.001**
Gender:				**<0.001**
Female	52 (91.2)	20 (62.5)	52 (91.2)	
Educational level:				**0.027**
Primary studies	17 (29.8)	19 (59.4)	18 (31.6)	
Secondary studies	33 (57.9)	8 (25.0)	32 (56.1)	
University	7 (12.3)	5 (15.6)	7 (12.3)	
Civil status:				**0.006**
Single	18 (31.6)	4 (12.5)	26 (45.6)	
Married/with stable partner	35 (61.4)	20 (62.5)	25 (43.9)	
Separated/Widower	4 (7.0)	8 (25.0)	6 (10.5)	
Work activity:				**<0.001**
Student	12 (21.1)	5 (15.6)	13 (22.8)	
Unemployed	5 (8.8)	4 (12.5)	20 (35.1)	
Sick leave/Pensioner	3 (5.3)	11 (34.4)	8 (14.0)	
Active	37 (64.9)	12 (37.5)	16 (28.1)	
Coexistence:				**0.004**
Single	4 (7.0)	3 (9.4)	11 (19.3)	
Family of origin	13 (22.8)	4 (12.3)	22 (38.6)	
Own family	40 (70.2)	25 (78.1)	24 (42.1)	

Abbreviations: BPD, borderline personality disorder; *n* (%), absolute frequency (relative frequency); $\bar{X}$ ± SD, mean ± standard deviation. Bolt characters represents statistical significative values. *p*-value of categorical variables: test Chi-Square, and the continuous variables: ANOVA test. Bold numbers represent statistically significant values.

**Table 2:** Group comparison between relatives with borderline personality disorder and controls in the “movie for the assessment of social cognition” (MASC).

MASC Subscores	MASC Sum Score	Relatives	BPD	Controls	*p*-Value *
	$\bar{X}$ ± SD	$\bar{X}$ ± SD	$\bar{X}$ ± SD	$\bar{X}$ ± SD	
	*n* = 146	*n* = 32	*n* = 57	*n* = 57	
MASC subscore emotion	9.3 ± 2.2	8.4 ± 2.1	9.1 ± 2.4	9.9 ± 1.9	**0.009**
MASC subscore thoughts	3.0 ± 0.9	2.8 ± 1.0	3.0 ± 0.9	3.2 ± 0.8	**0.029**
MASC subscore intentions	9.0 ± 1.8	8.5 ± 2.0	8.7 ± 1.6	9.6 ± 1.6	**0.003**

Abbreviations: $\bar{X}$ ± SD, mean ± standard deviation; BPD, borderline personality disorder; MASC, Movie for the Assessment of Social Cognition; *p*-value *: ANOVA test; Bold numbers represent statistically significant values.

**Table 3:** Post-hoc analysis with the Bonferroni Correction (*p*-values) for the scores of the scales applied in the three study groups.

	Relatives vs. Controls	BPD vs. Controls	BPD vs. Relatives
MASC subscore emotion	0.009	0.148	**0.534**
MASC subscore thoughts	0.031	0.254	**0.784**
MASC subscore intentions	0.013	0.012	**1.000**

Abbreviations: BPD, borderline personality disorder. Bold numbers represent statistically significant values.

**Table 4:** Multivariate analysis of the subscore intentions used in our patients, their relatives and controls (coefficients with their 95% confidence intervals).

Subscore Intentions						
Variable	Primary School		Secondary School		University	
	*n* = 54		*n* = 73		*n* = 19	
	β (95% IC)	*p*-Value	β (95% IC)	*p*-Value	β (95% IC)	*p*-Value
Category						
Controls	Ref.		Ref.		Ref.	
Relatives	−1.40 (−2.66; −0.14)	**0.03**	0.23 (−0.97; 1.43)	0.708	−2.20 (−3.93; −0.47)	**0.016**
BPD	−1.12 (−2.47; 0.24)	0.103	**−1.27 (−2.08; −0.46**)	**0.002**	−1.95 (−3.78; −0.12)	**0.038**
Coexistence						
Family of origin	Ref.	-	Ref.		Ref.	
Own family	−1.40 (−2.72; −0.07)	**0.039**	0.37 (−0.43; 1.18)	0.354	−1.08 (−3.78; 1.00)	0.284
Single	−0.68 (−2.44; 1.80)	0.440	1.04 (−0.21; 2.29)	0.101	−2.78 (−5.88; 0.31)	0.0743

Bold numbers represent statistically significant values.

**Table 5:** Multivariate analysis of the MASC subscores used for our patients (emotion and thought), their relatives and controls (coefficients with their 95% confidence intervals).

Variable	Subscore Emotion		Subscore Thoughts	
	*n* = 146		*n* = 146	
	β (95% IC)	*p*-Value	β (95% IC)	*p*-Value
Category				
Controls	Ref.		Ref.	
Relatives	−1.11 (−2.06; −0.16)	**0.021**	−0.15 (−0.57; 0.27)	0.487
BPD	−0.78 (−1.56; −0.16)	**0.047**	−0.17 (−0.47; 0.13)	0.267
Primary school	Ref.		Ref.	
Secondary school	1.22 (0.45; 1.99)	**0.002**	0.35 (0.06; 0.65)	**0.020**
University	1.67 (0.55; 2.78)	**0.002**	0.32 (−0.11; 0.75)	0.142
Married/with stable parther	-	-	Ref.	
Single	-	-	−0.67 (−0.99; −0.35)	**<0.001**
Separated/widower	-	-	−0.36 (−0.80; 0.07)	0.103
Age (years)	-	-	−0.02 (−0.03; 0.00)	**0.013**

Notes: BPD, borderline personality disorder; MASC, movie for the assessment of social cognition; all the coefficients were adjusted by educational level. Bold numbers c represent statistically significant values.

## Data Availability

The data is not available to third parties, as our informed consent provided to the participants indicates that the data would not be disclosed to third parties outside the study.
